# Differential Responses to a Visual Self-Motion Signal in Human Medial Cortical Regions Revealed by Wide-View Stimulation

**DOI:** 10.3389/fpsyg.2016.00309

**Published:** 2016-03-04

**Authors:** Atsushi Wada, Yuichi Sakano, Hiroshi Ando

**Affiliations:** ^1^Multisensory Cognition and Computation Laboratory, Universal Communication Research Institute – National Institute of Information and Communications TechnologyKyoto, Japan; ^2^Brain Networks and Communication Laboratory, Center for Information and Neural Networks, National Institute of Information and Communications Technology, Osaka UniversityOsaka, Japan; ^3^Graduate School of Frontier Biosciences, Osaka UniversityOsaka, Japan

**Keywords:** ego-motion, optic flow, forward vection, fMRI, multi-voxel pattern analysis, motion-in-depth, binocular disparity

## Abstract

Vision is important for estimating self-motion, which is thought to involve optic-flow processing. Here, we investigated the fMRI response profiles in visual area V6, the precuneus motion area (PcM), and the cingulate sulcus visual area (CSv)—three medial brain regions recently shown to be sensitive to optic-flow. We used wide-view stereoscopic stimulation to induce robust self-motion processing. Stimuli included static, randomly moving, and coherently moving dots (simulating forward self-motion). We varied the stimulus size and the presence of stereoscopic information. A combination of univariate and multi-voxel pattern analyses (MVPA) revealed that fMRI responses in the three regions differed from each other. The univariate analysis identified optic-flow selectivity and an effect of stimulus size in V6, PcM, and CSv, among which only CSv showed a significantly lower response to random motion stimuli compared with static conditions. Furthermore, MVPA revealed an optic-flow specific multi-voxel pattern in the PcM and CSv, where the discrimination of coherent motion from both random motion and static conditions showed above-chance prediction accuracy, but that of random motion from static conditions did not. Additionally, while area V6 successfully classified different stimulus sizes regardless of motion pattern, this classification was only partial in PcM and was absent in CSv. This may reflect the known retinotopic representation in V6 and the absence of such clear visuospatial representation in CSv. We also found significant correlations between the strength of subjective self-motion and univariate activation in all examined regions except for primary visual cortex (V1). This neuro-perceptual correlation was significantly higher for V6, PcM, and CSv when compared with V1, and higher for CSv when compared with the visual motion area hMT+. Our convergent results suggest the significant involvement of CSv in self-motion processing, which may give rise to its percept.

## Introduction

Sensing how our bodies move in relation to our surrounding environment is vital for spatial orientation and navigation (Gibson, 1986). Visual optic-flow is known to evoke a strong perception of self-motion, even in the absence of corresponding vestibular inputs (Brandt et al., 1973).

Human neuroimaging investigations of visual self-motion have largely focused on neural responses to optic-flow (de Jong et al., 1994; Brandt et al., 1998; Previc et al., 2000; Rutschmann et al., 2000; Peuskens et al., 2001; Wiest et al., 2001; Kleinschmidt et al., 2002; Deutschländer et al., 2004; Kovács et al., 2008; Wall and Smith, 2008; Cardin and Smith, 2010, 2011; Pitzalis et al., 2010, 2013; Becker-Bense et al., 2012; Cardin et al., 2012; Arnoldussen et al., 2013). These studies have described optic-flow sensitivity in multiple cortical regions, including the human medial superior temporal area (hMST) in the visual motion complex hMT+ (Peuskens et al., 2001; Smith et al., 2006), the cortical vestibular area in the parieto-insular vestibular cortex (PIVC; Cardin and Smith, 2010), and the ventral intraparietal area (VIP; Peuskens et al., 2001; Wall and Smith, 2008; Cardin and Smith, 2010), which correspond to results from several monkey studies (e.g., MST: Saito et al., 1986; Tanaka et al., 1986, 1989; Tanaka and Saito, 1989; Duffy and Wurtz, 1991a,b, 1995; Graziano et al., 1994; Lagae et al., 1994; Page and Duffy, 2003; PIVC: Akbarian et al., 1988; VIP: Schaafsma and Duysens, 1996; Schaafsma et al., 1997; Bremmer et al., 2002).

Recently, a number of human studies have identified optic-flow sensitive regions in the medial cortical wall, including visual area V6 (Cardin and Smith, 2010; Pitzalis et al., 2010), the precuneus motion area (PcM; Cardin and Smith, 2010), and the cingulate sulcus visual area (CSv; Wall and Smith, 2008; Cardin and Smith, 2010). Several different primary roles have been suggested for these regions in self-motion processing, especially the CSv. Stimuli with a stereoscopic depth associated with self-motion have been found to induce responses in V6 (Cardin and Smith, 2011; Arnoldussen et al., 2013) and the CSv (Arnoldussen et al., 2013). Fischer et al. (2012) reported that the CSv was better able to integrate eye movements with retinal motion than was V5/MT or the MST. The CSv and VIP have been found to exhibit selectivity to changing heading direction (Furlan et al., 2013). The CSv has also been reported to have vestibular sensitivity, along with the hMST and PIVC (Smith et al., 2012). These studies suggest differential roles for the medial optic-flow regions in visual self-motion processing. However, electrophysiological evidence is minimal, and thus, the way in which these regions differ in representing and processing visual self-motion signals (i.e., optic-flow) remains elusive.

Another unresolved issue in self-motion estimation concerns the way in which subjective perception (vection) arises from the neural processing of optic-flow. Instead of simply examining the optic-flow response, a number of neuroimaging studies have specifically investigated the neural correlates of self-motion perception (Brandt et al., 1998; Kleinschmidt et al., 2002; Kovács et al., 2008; Becker-Bense et al., 2012). Despite these attempts, the results have been somewhat inconsistent. Kleinschmidt et al. (2002) and Kovács et al. (2008) compared activation elicited by vection and non-vection conditions during continuous optic-flow stimulation, and identified a widely distributed cortical response to vection. However, the only region activated in both conditions was the hMT+, which was activated in response to vection in Kovács et al. (2008) and inhibited in Kleinschmidt et al. (2002). Other studies have quantitatively assessed the correlation between neural activation and subjective measures of vection, and each found different regions to be significantly correlated: the medial parieto-occipital region in Brandt et al. (1998) and the parahippocampal region in Becker-Bense et al. (2012). These inconsistencies might be related to differences in the naturalistic viewing conditions used to elicit self-motion perception, such as large field-of-view stimulation (Kleinschmidt et al., 2002; Becker-Bense et al., 2012) and stereoscopic depth cues (Kovács et al., 2008), because these are known to affect subjective vection strength (large field-of-view: Nakamura and Shimojo, 1998; Tarita-Nistor et al., 2006; stereoscopic depth cues: Palmisano, 1996, 2002).

Here, we investigated the neural representation of the visual self-motion signal. Specifically, we focused on areas V6, PcM, and CSv—three medial regions associated with optic-flow. We also measured activity in the hMT+, PIVC, and a control area (V1) for comparison. We conducted an fMRI experiment with a custom-developed fMRI-compatible visual presentation system. Our aim was to induce robust self-motion processing via a naturalistic viewing condition (i.e., a wide-view stereoscopic display) while measuring associated neural activity. We used multi-voxel pattern analysis (MVPA) in combination with conventional univariate analysis to examine spatial response patterns and the univariate activation profile, respectively. MVPA is thought to be able to detect more detailed neural representations than conventional univariate analyses (see Norman et al., 2006 for review). We measured responses to random-dot stimuli that were either static, in random motion, or in coherent motion that simulated forward self-motion (i.e., radially expanding optic-flow). We hypothesized that if a brain region contains a representation specific to visual self-motion signals, it would exhibit both optic-flow-selective univariate activation and a multi-voxel response pattern. The multi-voxel response pattern would discriminate coherent motion from random motion and no-motion, but would not discriminate random motion and no-motion stimuli from each other. We included stereoscopic and non-stereoscopic conditions to assess the effect of binocular cues on perception of self-motion. We systematically varied stimulus size across four levels. The purpose of this was to examine the presence of visual spatial representation (e.g., retinotopy) in the target region. We assumed that if a region represents global optic-flow induced by self-motion instead of local optic-flow generated by object motion, it would show a multi-voxel spatial response pattern insensitive to the coverage and location of optic-flow in the visual field (i.e., no clear visual spatial representation such as retinotopy) as long as an identical self-motion profile (e.g., forward self-motion) is signaled. We also measured neuro-perceptual correlations of subjective self-motion strength, which enabled us to assess the involvement of each medial brain region in generating the perception of self-motion in response to optic-flow stimuli. Here, varying the stimulus size had the additional purpose of largely differentiating subjective self-motion strengths to allow an effective investigation of the neuro-perceptual correlation.

## Materials and Methods

### Subjects

Two of the authors and 11 naive volunteers (seven males and six females, 20–44 years of age, mean age 34.5 years) participated in the main fMRI experiment. Of these 13 subjects, the two authors and seven volunteers (five males and four females, 23–44 years of age, mean age 35.7 years) further participated in the localizer and psychophysics experiments. All subjects were healthy, with normal or corrected-to-normal vision, and no history of neurological disorders. All subjects had experience with remaining still and maintaining fixation during fMRI experiments. The subjects provided written informed consent regarding the experimental procedures, which were approved by the ATR Human Subject Review Committee (Keihanna Science City, Kyoto).

### Apparatus

**FIGURE 1:**
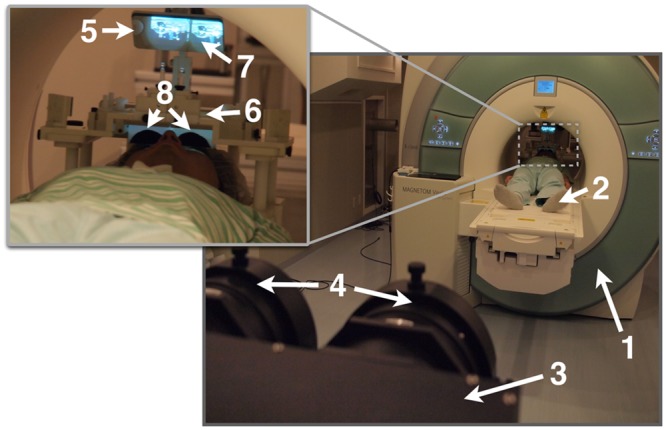
**Photograph of apparatus setup for wide-view stereoscopic visual stimulation inside a MRI scanner.** (1) MRI scanner, (2) subject, (3) video projectors in electromagnetically shielded box, (4) telescopic lenses for long-distance video projection, (5) front surface mirror, (6) projection screen, (7) stereoscopic visual image on the projection screen viewed via the mirror, and (8) wide-view binocular eyepiece lenses.

We used a 3-T MR scanner (Siemens TIM Trio; Siemens Medical Systems, Erlangen, Germany) equipped with a 12-channel head coil to acquire functional and anatomical magnetic resonance (MR) images. For the wide-view stereoscopic stimulation inside the scanner, we used a custom developed MRI-compatible visual presentation system (**Figure 1**). The dichoptic images were projected into the scanner bore using two video projectors (JVC, DLA-HD11K, Yokohama, Japan, 60 Hz, 1920 × 1080 resolution) covered with radio frequency (RF) shielding and a pair of custom-designed long distance telescope lenses. The projection unit was positioned inside the MRI room near the RF-shielded wall. The video image for each eye was separately projected onto a translucent back-projection screen located on a pair of custom-designed eyepiece lenses. These were assembled as a single unit and attached to the head coil, which we operated in half-coil mode. The optical profile of the lenses and their assembly with the screen was designed to simulate an infinite viewing distance with no vergence-accommodation conflict. The eyepiece lenses were positioned in front of the subjects, in close proximity to their eyes, which enabled a wide-view (100° × 67.7°) stereoscopic visual stimulation. The system was designed to be safe for use inside 3T-MR scanners, as the projection component was placed outside the five-gauss line, and the head-coil component contained no ferromagnetic materials. MRI artifacts caused by the system were in the permissive range.

### Visual Stimuli and Experimental Procedures

In all experiments, subjects fixated on a square (0.5° × 0.5°) in the center of the screen when it was present. When it was not present, they fixated on the center of the screen.

#### Main fMRI Experiment

**FIGURE 2:**
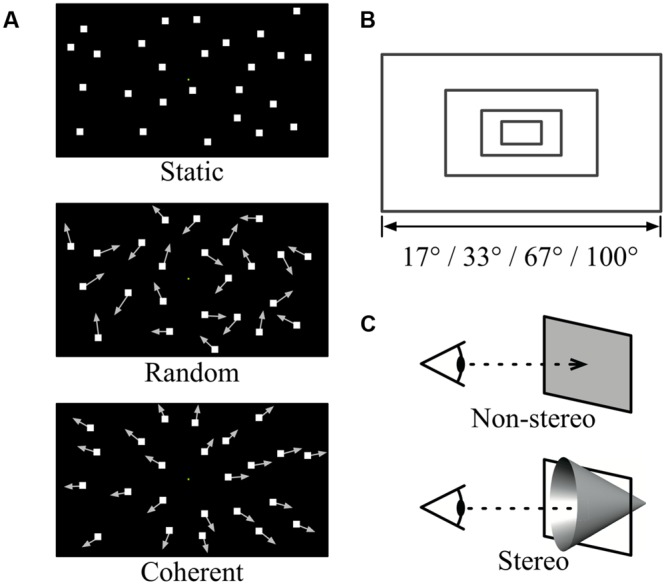
**Schematic illustration of the stimulus conditions.** Dynamic random dots were created by combining three factors: **(A)** motion pattern, **(B)** stimulus size, and **(C)** stereo factors.

The visual stimuli in the main fMRI experiment were random dot sequences that had specific motion patterns, sizes, and stereo factors (**Figure 2**). The dots were white squares (visual angle, 0.2° × 0.2°) on a black background. They had a lifetime of 167 ms with asynchronous refresh times. The dots were either static, in random motion, or in coherent motion (**Figure 2A**). In the coherent motion condition, a radial expansion of dots induced optic-flow that simulated forward self-motion. The velocity of each dot increased linearly as a function of eccentricity from the screen center, which ranged between 0.12 and 3.9°/s. The spatial distribution of dot speeds under the random motion condition was identical to that of the coherent motion condition, but the motion direction of each dot was randomized. The stimuli subtended horizontal viewing angles of 17, 33, 67, or 100°, and had an aspect ratio of 16:9 (**Figure 2B**). The total number of dots increased linearly with the area of the stimulus, up to a maximum of 800. We included stereo and non-stereo conditions (**Figure 2C**), designed based on Cardin and Smith (2011). In the stereo condition, a binocular disparity-defined depth gradient was embedded in the stimulus and the fixation square had a crossed disparity of 1.0° relative to the screen. Additionally, the depth changed from far to near (varying by ±3.77°) with absolute disparity set as a linear function of eccentricity. In the non-stereo condition, the depth of the entire stimulus was uniformly set to have zero disparity relative to the infinite screen. There were 24 experimental conditions, which comprised all possible combinations of the three factors.

We used a rapid event-related design for the main fMRI experiment. The stimulus duration was 3 s and the inter-stimulus interval (ISI) varied between 2 and 8 s according to a Poisson distribution (mean ISI = 3.7 s). Each of the 24 types of random-dot stimuli was presented twice (in total, 48 trials) during each scanning run, which started and ended with a 10 s period in which the screen was blank. The stimulus order was counterbalanced across scanning runs and subjects. Eight runs were performed for each subject, and each run lasted 340 s. Subjects were instructed to attend to the stimuli during the run. To ensure fixation, they were asked to count and report color changes to the fixation square during each run.

#### Localizer Experiment

We functionally identified the hMT+ using a motion localizer procedure (Huk et al., 2002). The stimuli consisted of 27-s blocks of moving dots, alternating with 9-s blocks of static dots. The dots were white squares of equal size (0.25° × 0.25°) on a black background. The moving dots traveled toward and away from the fixation point (8°/s), altering their direction every second. Two runs were performed for each subject, and each run consisted of eight cycles of alternating motion and stationary pattern blocks.

We functionally identified the locations of V6, PcM, and CSv using a flow-fields localizer procedure (Pitzalis et al., 2010). The stimuli consisted of 16-s blocks of coherent dot-field motion alternating with scrambled motion. The dots were white squares of equal size (0.4° × 0.4°) on a black background. The coherent dot-field motion was chosen randomly from a continuum of patterns including expansion to outward spiral, rotation, inward spiral, and contraction, and the pattern was refreshed every 500 ms. The center of movement was jittered from flow to flow. In the scrambled-motion block, the spatial velocity distribution was identical to that in the coherent dot field-motion block, although the motion direction of each dot was randomized. Six runs were performed for each subject, and each run consisted of eight cycles of alternating coherent dot field and random motion-pattern blocks.

#### Psychophysical Experiment

We used the magnitude estimation method to measure the perception of visual self-motion induced by the stimuli used in the main fMRI experiment. This measurement was conducted in a separate session from the main fMRI experiment, although we used the same visual presentation system with the same MR scanner. The standard stimulus (SS) was the coherent motion in the 100° stereo condition. Subjects rated the subjective strength of perceived self-motion for each of the 24 stimuli compared with the SS, which appeared just before the presentation of each target stimulus. The stimulus duration was identical to that in the main fMRI experiment: 3 s for both the target stimulus and the SS stimuli. A score of 0 corresponded to a complete lack of perceived self-motion while a score of 10 corresponded to maximal perceived self-motion in response to the SS.

### Magnetic Resonance Imaging Data Acquisition

We acquired single-shot echo-planar imaging (EPI) images for the functional scanning runs, with the following imaging parameters: repetition time (TR), 2,000 ms; echo time (TE), 30 ms; flip angle (FA), 80°; voxel size, 3 mm × 3 mm × 3 mm; matrix size, 64 × 64; and 30 slices aligned to the anterior–posterior commissure (AC–PC) line covering the entire occipital cortex and the posterior parts of the temporal and parietal cortices.

We acquired a pair of T1-weighted anatomical images (magnetization-prepared rapid gradient echo) for each subject, which was used for spatial normalization, cortical surface extraction, and visualization. The imaging parameters were as follows: TR, 2,250 ms; TE, 3.06 ms; FA, 9°; voxel size, 1 mm × 1 mm × 1 mm; matrix size, 64 × 64; and 192–208 sagittal slices covering the whole head.

To aid accurate mapping between the anatomical and functional images, we acquired T2-weighted images (turbo spin echo) with high in-plane resolution for each subject. The field of view, slice thickness, number of slices, and slice orientation of these images were identical to those of the functional EPI images. Other imaging parameters were as follows: TR, 6,000 ms; TE, 57 ms; FA, 160°; voxel size, 0.75 mm × 0.75 mm × 3 mm; and matrix size, 256 × 256.

### Data Analysis

#### Statistical Parametric Mapping Analysis

We used SPM8 software (Wellcome Trust Centre for Neuroimaging, London, UK) to conduct statistical parametric mapping (SPM) analysis on the data from all 13 subjects. Using the functional EPI images, we applied slice-timing correction, motion correction, spatial normalization to the Montreal Neurological Institute (MNI) stereotaxic space, and spatial smoothing using a 3D Gaussian kernel (3-mm full width at half maximum). We performed SPM analysis based on the general linear model (GLM). Each of the 24 experimental conditions was modeled as an independent GLM regressor for each run, the time course of which was derived from the corresponding stimulus presentation time periods for each subject. The six-dimensional head-motion correction parameters were also included as regressors in the model to dissociate subject motion-induced components. A set of beta-coefficient images corresponding to the regressors were generated via individual-level GLM analysis, and these were entered into a subsequent group-level analysis that examined the following contrasts: (1) coherent motion selective response (conjunction of coherent vs. static and coherent vs. random), (2) general motion-sensitive response (conjunction of random vs. static and coherent vs. static), (3) stimulus-induced response (stimulus vs. blank) for each stimulus size, and (4) stereo-induced response (stereo vs. non-stereo). We applied multiple-comparison correction to the resulting maps with an uncorrected height threshold of *p* < 0.001 at the peak level and a cluster-level false discovery rate (FDR) set at *p* < 0.05. For ease of visualization, the significantly activated clusters were rendered on an average cortical surface ‘fsaverage’ provided in the FreeSurfer software^1^ (Dale et al., 1999; Fischl et al., 1999).

#### Region of Interest Localization

**FIGURE 3:**
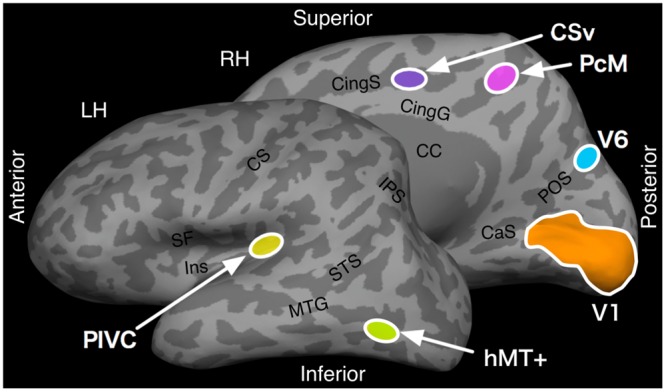
**Anatomical locations of the regions of interest (ROIs).** The locations of cortical areas V1, hMT+, V6, PcM, CSv, and PIVC are shown on the inflated surface of the cortex. CS, central sulcus; SF, Sylvian fissure; Ins, insular cortex; IPS, intra-parietal sulcus; STS, superior temporal sulcus; MTG, middle temporal sulcus; CaS, calcarine sulcus; POS, parieto-occipital sulcus; CingG, cingulate gyrus; CingS, cingulate sulcus; CC, corpus callosum; LH, left hemisphere; RH, right hemisphere.

We performed ROI localization using SPM8 in MATLAB (The MathWorks, Natick, MA, USA) and the MarsBaR MATLAB toolbox^2^ (Brett et al., 2002) for the nine subjects who participated in the localizer experiments. To identify the ROIs that we used in the subsequent univariate ROI and multi-voxel correlation analyses, we used a procedure that mirrored that of SPM analysis, except we did not conduct spatial smoothing or multiple-comparison corrections. We localized hMT+ by contrasting the motion and stationary conditions in the motion localizer. The significance threshold was set between *p* = 0.005 and *p* = 0.00005; the exact value was set to that which would delineate a distinct cluster corresponding to hMT+. The cortical regions V6, PcM, CSv, and PIVC were similarly identified by contrasting the coherent dot-field motion with the scrambled-motion conditions in the flow-fields localizer. The significance threshold was set at *p* = 0.001. We used the FreeSurfer software suites to identify the location of primary visual area V1. This was done via a cortical surface-based probability map, which was estimated for each subject. Furthermore, we used a three-step procedure to anatomically segregate V1 using its surface-based probabilistic map (Hinds et al., 2008): (1) cortical surface reconstruction using T1-weighted anatomical image, (2) surface-based registration to an average template using the gyral and sulcal patterns, and (3) estimation of V1 using the surface-based probabilistic map of V1. This V1 localization method has been shown to agree highly with retinotopic mapping results (Benson et al., 2011). The anatomical locations of these ROIs are illustrated on an inflated representation of the cortical hemispheres in **Figure 3**.

#### Univariate Region of Interest Analysis

We performed univariate ROI analysis using the MarsBaR toolbox. For each ROI, we extracted a ROI-averaged signal time course and applied GLM analysis; the models were identical to those used in the SPM analysis. The contrast estimates computed by GLM analysis for all 24 experimental conditions were then scaled to the blood-oxygen-level dependence (BOLD) signal changes, which we collected and averaged across the experimental runs and the subjects. The resulting ROI-averaged neural activation data were then entered into a three-way repeated-measures analysis of variance (ANOVA). We tested the statistical significance of the main effects and the interactions between Motion Pattern, Stimulus Size, and Stereo Factors for each ROI. For the main effects of Motion Pattern and Stimulus Size, each of which consisted of more than two levels, the degrees of freedom were adjusted using Greenhouse–Geisser correction if the deviance from sphericity was significant. We further conducted *post hoc* paired *t*-tests with Bonferroni–Holm adjustments, which allowed us to further assess the difference among the static, random motion, and coherent motion conditions that comprised the motion pattern factor. We used the R software ez package to run these statistical tests (R Foundation for Statistical Computing, Vienna, Austria).

#### Multi-voxel Pattern Analysis

We performed MVPA (see Norman et al., 2006 for review) to examine how the assessed regions differed in their multi-voxel response profile. MVPA can preserve and discriminate spatial response patterns that are otherwise lost by averaging responses across voxels within an ROI, as in the case of the univariate analysis.

Here, we adapted the method used by Furlan et al. (2013), which applied a linear support vector machine (SVM) to assess the multi-voxel response pattern in optic-flow sensitive regions. The basic idea was to test each ROI for whether the SVM, after being trained using part of the data, could use the information contained in the multi-voxel pattern to accurately predict the experimental condition under which the rest of each data sample was acquired. We used this procedure to classify each of the three experimental factors (Motion Pattern, Stimulus Size, and Stereo) to determine whether a corresponding neural representation existed. For example, three-class classification (coherent motion, random motion, or static conditions) was performed for the Motion Pattern factor to assess whether the resulting prediction accuracy was significantly higher than chance level. The detailed pattern of misclassification is illustrated by a confusion matrix depicting the SVM classifier’s actual predictions for data from each experimental condition. The rows and columns of the confusion matrix correspond to the original and predicted labels, respectively. The confusion matrix was also used to derive binary classification results for each of the possible pairs of Motion Pattern conditions (coherent vs. random motion, coherent motion vs. static, and random motion vs. static).

Before SVM classification, multi-voxel patterns for each ROI were obtained by performing voxelwise GLM analyses. The model used for these analyses was basically the same as that for the conventional univariate SPM analysis except that the regressors were modeled separately for each stimulus presentation trial. The GLM fitting was performed for each of the eight scan runs. We obtained 16 data samples (i.e., a vector of beta values comprising the ROI) for each of the 24 experimental conditions for each subject. To avoid a possible underestimation of prediction accuracy that might occur when applying SVM classification to data with a small number of voxels, we concatenated voxels across subjects using a validated procedure that we adapted from previous studies (Brouwer and Heeger, 2009; Furlan et al., 2013). The number of voxels was then equalized across ROIs by selecting 112 voxels from each ROI. This number was chosen because it was the number of all available voxels for the CSv, pooled across subjects, which was the smallest among all the assessed ROIs. Here, the voxels were selected by descending down the ranking based on average activation intensity across all experimental conditions. We also validated the influence of number of voxels on SVM classification by examining the prediction accuracy for each ROI as a function of the selected number of voxels, which used the same descending order of ranking based on average activation intensity (Furlan et al., 2013).

Prediction accuracy was computed by performing SVM classification with a leave-one-run-out cross validation method. More specifically, the data samples obtained from 7 out of 8 runs were used for training, and the remaining run was used for testing. This train-test split was repeated eight times so that each of the 8 runs was left out once, and the resulting 8 prediction accuracies were averaged. Finally, for each classification of each ROI, we performed a permutation test to estimate the statistical significance of the prediction accuracy. The null distribution of prediction accuracy was generated by running SVM classification on the same dataset, but randomly assigning permuted experimental conditions within each run. This random permutation was repeated 10000 times for each permutation test. The prediction accuracy obtained using the real data was considered significant when above the 95% percentile of the permutation distribution. We also conducted direct pairwise comparisons of prediction accuracy in V6, PcM, and CSv. The same permutation procedure was applied to assess the significance of the pairwise difference in prediction accuracy among the medial optic-flow regions. The classification was considered significant if the pairwise difference using real data was greater than 95% (9500) of the pairwise differences using permuted data. Additionally, prediction accuracies obtained from the binary classification of motion-pattern pairs and from the Stimulus Size classification for each motion pattern were similarly examined. This included direct pairwise comparisons of prediction accuracies among the different classifications (i.e., the three motion-pattern pairs in the binary classification or the three motion patterns in the Stimulus size classification) within each ROI. We applied permutation procedures identical to those described above. The significance level was corrected for multiple comparisons using Bonferroni–Holm’s method when performing pairwise comparisons among different ROIs or classifications. GLM analysis was performed with SPM8 and the MarsBaR MATLAB toolbox. The rest of the analyses (i.e., voxel concatenation across subjects, voxel selection, and SVM classification) were performed using MATLAB using the LIBSVM library for SVMs (Chang and Lin, 2011).

#### Psychophysical Analysis

We collected ratings of vection strength from the nine subjects who participated in the psychophysics experiment, and then entered these data into a three-way repeated-measures ANOVA. The degrees of freedom were corrected for deviance from sphericity (Greenhouse–Geisser) if necessary. Statistical significance for the main effects and the interactions between motion pattern, stimulus size, and stereo factors were determined. For the Motion Pattern factor, we performed *post hoc* paired *t*-tests with Bonferroni–Holm adjustment, which allowed us to further assess the differences among the static, random motion, and coherent motion conditions. We used the R software ez package to run these statistical tests.

#### Neuro-perceptual Correlation Analysis

We used custom MATLAB scripts to investigate the neuro-perceptual correlation between ROI-averaged neural activation and perceived self-motion strength. BOLD signal changes for all 24 experimental conditions were z-score normalized within each subject and then collected across the nine subjects that participated in the localizer experiments. We computed Pearson’s correlation coefficients between the BOLD signal change and the rating score. The correlation coefficients were then converted into a t-score and subjected to a Student’s *t*-test. The significance threshold was set at *p* < 0.05. We performed pairwise comparisons among the ROIs to test whether the two correlation coefficients significantly differed from one another. Here, the two correlation coefficients assessed in each pairwise test were not independent, because the corresponding computations shared the same rating-score data. Thus, we applied the method proposed in Cohen and Cohen (1983) to account for deviation from the assumption of independence. As we compared the correlation coefficients among ROIs, we applied Bonferroni correction based on the number of comparisons.

## Results

### SPM Analysis

**FIGURE 4:**
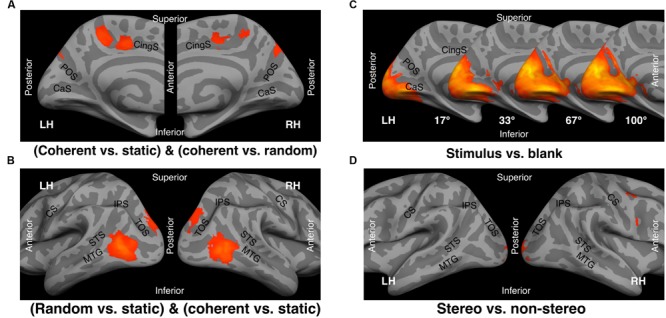
**Average *t*-statistical maps (13 subjects) displayed on the inflated averaged cortical surface.** Statistically significant clusters of contiguous activation were identified by SPM analysis (height thresholds: uncorrected *p* < 0.001 at peak level, *p* < 0.05 FDR-corrected at cluster level). **(A)** Coherent motion selective response (conjunction of coherent vs. static and coherent vs. random motion). **(B)** General motion-induced response (conjunction of random motion vs. static and coherent motion vs. static). **(C)** Stimulus induced response (stimulus vs. blank) for each of the four stimulus sizes. **(D)** Stereo-induced response (stereo vs. non-stereo). CaS, calcarine sulcus; POS, parieto-occipital sulcus; CingS, cingulate sulcus; CS, central sulcus; STS, superior temporal sulcus; MTG, middle temporal gyrus; IPS, intraparietal sulcus; TOS, transverse occipital sulcus; LH, left hemisphere; RH, right hemisphere.

Using SPM analysis, we identified 22 clusters of activation that met the criteria for cluster-level FDR control (**Table 1**). The coherent motion-selective contrast (conjunction of coherent vs. static and coherent vs. random) identified three bilateral pairs of clusters located in the medial wall, V6, PcM, and CSv (**Figure 4A**). The general motion-sensitive contrast (conjunction of random vs. static and coherent vs. static) identified two bilateral pairs of clusters, hMT+ and V3A (**Figure 4B**). The contrast for stimulus-induced response (stimulus vs. blank) for each stimulus size identified a bilateral pair of clusters that covered broad regions in the visual cortex along the ventral and dorsal visual pathways (**Figure 4C**). The lateral geniculate nuclei (LGN) were also identified as part of this cluster (right LGN in the 67° condition and bilaterally in the 100° condition) or as separate clusters (bilaterally in the 33° condition and in the left LGN in the 67° condition). The total activated volume of the stimulus-induced response increased with stimulus size (**Table 1**, C-1 to C-4). The contrast for stereo-induced response (stereo vs. non-stereo) identified a bilateral pair of clusters in the early visual cortex, a cluster in each right inferior partial lobule, and a cluster in the right precentral gyrus (**Figure 4D**).

**Table 1:** Cortical and subcortical regions identified by SPM analysis.

Region name	Laterality	Peak location			Peak val.	Size
		*x*	*y*	*z*	(Z-score)	(mm^3^)
**(A) Coherent motion selective response**					
Cingulate sulcus (CSv)	L	-12	-22	42	6.03	848
	R	10	-22	48	4.69	648
Precuneus (PcM)	L	-14	-42	52	5.99	1088
	R	18	-44	56	4.17	280
Parieto-occipital sulcus (V6)	L	-20	-82	46	5.36	1048
	R	18	-72	38	4.50	1488
**(B) General motion-induced response**					
Middle temporal gyrus (hMT+)	L	-40	-70	6	infinite	5608
	R	44	-72	4	infinite	5464
Transverse occipital sulcus (V3A)	L	-16	-90	26	5.36	2384
	R	24	-84	40	4.55	3576
**(C-1) Stimulus-induced response (17°)**					
Occipital cortex	LR	8	-78	-4	infinite	85688
**(C-2) Stimulus-induced response (33°)**					
Occipital cortex	LR	-6	70	0	infinite	105480
Thalamus (LGN)	L	-20	-30	-2	6.36	656
	R	24	-28	0	5.14	320
**(C-3) Stimulus-induced response (67°)**					
Occipital cortex and right thalamus	LR	2	-74	20	infinite	126880
Left thalamus (LGN)	L	-18	-30	-2	7.35	928
**(C-4) Stimulus-induced response (100°)**					
Occipital cortex and thalamus	LR	8	-78	-4	infinite	144584
**(D) Stereo-induced response**					
Calcarine sulcus and lingual gyrus	L	-16	-94	-8	6.44	3176
	R	18	-88	-2	7.31	3880
Intra-parietal sulcus (AIP)	R	46	-40	54	4.63	840
Precentral gyrus	R	52	6	30	4.52	360
	R	40	-4	50	4.12	384

*We identified clusters of statistically significant contiguous activation (height thresholds: uncorrected *p* < 0.001 at peak level, *p* < 0.05 FDR-corrected at cluster level). The coordinate of the peak location of each cluster is provided in MNI stereotaxic space.*

### Univariate ROI Analysis

**FIGURE 5:**
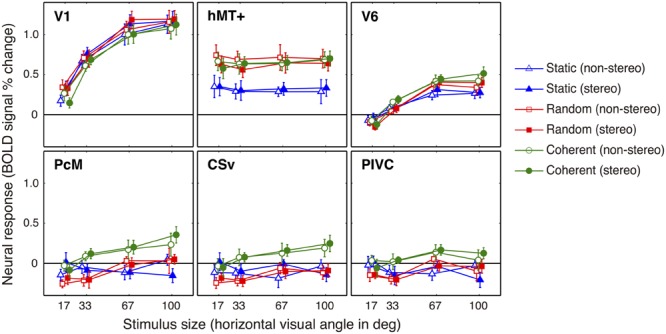
**Stimulus-induced response profiles for ROIs.** The mean activation in a ROI was averaged across nine subjects for each of the 24 conditions. Error bars indicate SEMs.

The response profiles for the ROI-averaged univariate neural activation for each ROI are shown in **Figure 5**, and the effects of the experimental factors are summarized in **Table 2**. We identified a significant main effect of Motion Pattern in all assessed regions (*F*_2,16_ = 10.4, *p* = 0.001; hMT+: *F*_2,16_ = 30.1, *p* = 0.000; V6: *F*_2,16_ = 16.8, *p* = 0.000; PcM: *F*_2,16_ = 18.3, *p* = 0.000; CSv: *F*_1.15,9.19_ = 19.8, *p* = 0.001; and PIVC: *F*_2,16_ = 7.77, *p* = 0.004), and the subsequent *post hoc* analysis revealed differential motion-pattern selectivity among these regions. Coherent motion selectivity was found in V6, PcM, CSv, and PIVC—activation was significantly larger in response to coherent motion than to random motion or no-motion, and responses to random motion and no-motion did not differ from each other. Additionally, the response to the random motion was significantly lower than to no-motion in the CSv, a unique profile among all assessed regions. The hMT+ exhibited general motion-sensitivity in which the neural responses to coherent and random motion were comparable and significantly larger than the response to the static condition. Furthermore, the control region V1 showed a significantly larger response to the random motion condition than to the static condition. The main effect of Stimulus Size was significant in all assessed regions except the hMT+ (V1: *F*_1.38,11.0_ = 94.8, *p* = 0.000; V6: *F*_1.43,11.4_ = 48.0, *p* = 0.000; PcM: *F*_3,24_ = 5.95, *p* = 0.003; CSv: *F*_3,24_ = 3.25, *p* = 0.040; PIVC: *F*_3,24_ = 3.92, *p* = 0.021). Among these regions, V6 (*F*_6,48_ = 4.66, *p* = 0.001) and PcM (*F*_6,48_ = 2.40, *p* = 0.042) showed a significant interaction between Motion Pattern and Stimulus Size, and CSv showed a tendency for an interaction (*F*_6,48_ = 2.02, *p* = 0.081). The main effect of Stereo Factor was not significant in any of the assessed regions, although its interaction with Motion Pattern was significant in V1 (*F*_2,16_ = 4.70, *p* = 0.025) and hMT+ (*F*_2,16_ = 4.39, *p* = 0.030).

**Table 2:** The effects of experimental factors on ROI-averaged neural responses.

ROI	V1	hMT+	V6	PcM	CSv	PIVC
**Main effect**						
Motion pattern	0.001^∗∗^	0.000^∗∗∗^	0.000^∗∗∗^	0.000^∗∗∗^	0.001^∗∗^	0.004^∗∗^
	(R > C)	(C > S, R > S)	(C > R, C > S)	(C > R, C > S)	(C > S > R)	(C > R, C > S)
Stimulus size	0.000^∗∗∗^	0.702	0.000^∗∗∗^	0.003^∗∗^	0.040^∗^	0.021^∗^
Stereo	0.150	0.465	0.223	0.688	0.255	0.831
**Interaction**						
Motion pattern × stimulus size	0.861	0.953	0.001^∗∗∗^	0.042^∗^	0.081^+^	0.112
Motion pattern × stereo	0.025^∗^	0.030^∗^	0.951	0.640	0.635	0.856
Stimulus size × stereo	0.449	0.670	0.253	0.826	0.689	0.893
All three factors	0.257	0.998	0.668	0.224	0.429	0.199

*We used a three-way repeated-measures ANOVA to examine the main effects and interactions between Motion Pattern, Stimulus Size, and Stereo Factor with respect to ROI-averaged neural responses. For the main effect of Motion Pattern, we performed *post hoc* paired *t*-tests with Bonferroni–Holm adjustment to further assess the pairwise differences between conditions. Statistical significance is expressed in terms of *p*-values. S: static; R: random motion; C: coherent motion. ^+^*p* < 0.1, ^∗^*p* < 0.05, ^∗∗^*p* < 0.01, ^∗∗∗^*p* < 0.001.*

### Results of the Multi-voxel Pattern Analysis

**FIGURE 6:**
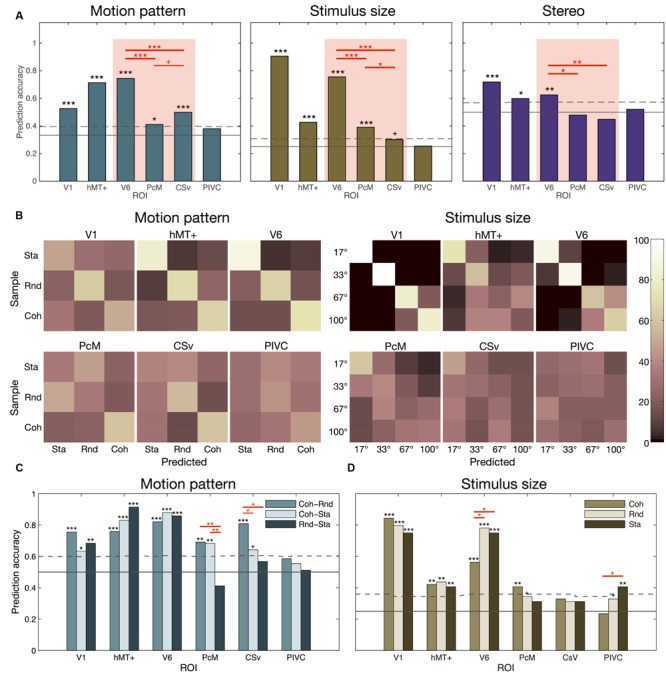
**Results of the multi-voxel pattern analysis. (A)** Classification results for each experimental factor. The SVM classification of all experimental conditions comprising the corresponding factor was performed for each ROI. Red lines indicate statistically significant differences in prediction accuracy between ROIs. **(B)** Confusion matrices showing the classification results for motion pattern and stimulus-size factors. The ratio (percent) of the predicted labels assigned to each experimental condition is represented by the color saturation of each cell in the matrix. **(C)** Results of the binary classification for each paired-motion pattern. Red lines indicate significant differences between binary classifications. **(D)** Results of the stimulus-size classification when limiting data to each motion pattern. Red lines indicate significant differences between motion pattern conditions. **(A,C,D)** The symbols above each bar indicate the statistical significance of the prediction accuracy when compared against chance level derived from randomly permuted data. Multiple comparison correction was applied when comparing the prediction accuracy between different classifications (depicted by red lines). The solid and broken horizontal lines represent the theoretical chance level and the 5% critical thresholds computed by a permutation test, respectively. ^+^*p* < 0.1; ^∗^*p* < 0.05; ^∗∗^*p* < 0.01; ^∗∗∗^*p* < 0.001.

The SVM classification results for Motion Pattern, Stimulus Size, and Stereo Factors are shown in **Figure 6A**. For Motion Pattern classification, all the assessed regions except for PIVC showed prediction accuracy that was significantly above chance level (V1: 0.526, *p* = 0.000; hMT+: 0.714, *p* = 0.000; V6: 0.745, *p* = 0.000; PcM: 0.411, *p* = 0.000; CSv: 0.500, *p* = 0.000). Prediction accuracy for V6 was significantly higher than for either PcM (*p* = 0.000) or CSv (*p* = 0.000). CSv tended to have a higher prediction accuracy than PcM (*p* = 0.055). For Stimulus Size classification, prediction accuracy was significantly above chance level for V1 (0.906, *p* = 0.000), hMT+ (0.427, *p* = 0.000), V6 (0.755, *p* = 0.000), and PcM (0.391, *p* = 0.000) and approached significance in CSv (0.302, *p* = 0.059). Prediction accuracy was significantly higher for V6 than for either PcM (*p* = 0.000) or CSv (*p* = 0.000). Similarly, accuracy for PcM was higher than for CSv (*p* = 0.030). For Stereo Factor classification, prediction accuracy was significantly higher than chance level in V1 (0.719, *p* = 000), hMT+ (0.599, *p* = 0.010), and V6 (0.625, *p* = 0.001). Prediction accuracy was significantly higher for V6 than for either PcM (*p* = 0.015) or CSv (*p* = 0.008). We also assessed the robustness of these results to the number of features (i.e., voxels) used in the SVM classification. The result of this procedure verified that the prediction accuracy became saturated as the number of voxels approached to the maximum of 112 (Supplementary Figure S2).

Patterns of misclassifications are illustrated by confusion matrices for Motion Pattern and Stimulus Size factors, each of which consisted of more than two experimental conditions (**Figure 6B**). The interpretation of a confusion matrix is as follows. For a fully successful classification, the data samples originally acquired for each label (i.e., a row in the confusion matrix) are all classified into the correct predicted label (i.e., the diagonal element within the row), which results in a matrix with its diagonal elements being 100% (white) and the others 0% (black). In contrast, for a fully random classification, the data samples for each original label (a row in the confusion matrix) are evenly assigned to each of the predicted labels (each element in the row), which results in a confusion matrix with a uniform value (pink) assigned to every element. The confusion matrices for Motion Pattern classification in hMT+ and V6 showed high values in their diagonal elements, which indicates successful classification for each motion pattern. PcM, in contrast, showed relatively accurate classification only for coherent motion. Meanwhile, CSv exhibited relatively accurate classification not only for coherent motion but also for random motion. Confusion matrices for Stimulus Size classification in V1 and V6 showed high values in the diagonal elements, indicating successful classification for each stimulus size, while those for CSv and PIVC showed relatively uniform values across each element, indicating relatively random classification. The confusion matrix for PcM showed an intermediate pattern between V6 and CSv, which implies that activity in PcM can partially discriminate stimulus size.

The results of the binary classifications for each pair of motion patterns are shown in **Figure 6C**. Prediction accuracies were significantly higher than chance level in all binary classifications obtained from V1 (coherent motion vs. random motion: 0.755, *p* = 0.000; coherent motion vs. static: 0.633, *p* = 0.013; random motion vs. static: 0.683, *p* = 0.002), hMT+ (accuracy = 0.759, *p* = 0.000, accuracy = 0.830, *p* = 0.000, and accuracy = 0.914, *p* = 0.000, respectively), and V6 (accuracy = 0.821, *p* = 0.000; accuracy = 0.879, *p* = 0.000; accuracy = 0.858, *p* = 0.000, respectively), which confirmed the profiles observed in the corresponding confusion matrices, indicating successful classification regardless of motion pattern. In contrast, PcM (coherent motion vs. random motion: 0.690, *p* = 0.003; coherent motion vs. static: 0.682, *p* = 0.003) and CSv (coherent motion vs. random motion: 0.809, *p* = 0.000; coherent motion vs. static: 0.642, *p* = 0.024) showed significantly higher prediction accuracy only for the binary classification involving coherent motion but not for random motion vs. static conditions. Prediction accuracy for PIVC was not significantly above chance for any binary classification. Pairwise comparisons of prediction accuracy among binary classifications within each ROI revealed significant differences between coherent vs. random motion and random motion vs. static classifications in the PcM (*p* = 0.002) and CSv (*p* = 0.012). Furthermore, prediction accuracy significantly differed between coherent motion vs. static and random motion vs. static classifications in the PcM (*p* = 0.003) and approached significance for the difference between coherent vs. random motion and coherent motion vs. static classifications in the CSv (*p* = 0.068).

The results of the Stimulus Size classifications using the data limited to each motion pattern are shown in **Figure 6D**. Limiting the data to any motion pattern resulted in a prediction accuracy that was significantly higher than chance level in V1 (coherent motion: 0.844, *p* = 0.000; random motion: 0.797, *p* = 0.000; static: 0.750, *p* = 0.000), hMT+ (coherent motion: 0.422, *p* = 0.002; random motion: 0.438, *p* = 0.001; static: 0.406, *p* = 0.003), and V6 (coherent motion: 0.562, *p* = 0.000; random motion: 0.781, *p* = 0.000; static: 0.750, *p* = 0.000). In contrast, limiting the data to either motion pattern resulted in no significant prediction accuracy in the CSv, although it approached significance in the case of coherent motion (0.328, *p* = 0.086). PcM showed an intermediate profile between V6 and CSv, in which prediction accuracy was significant when limiting data to the coherent motion condition (0.406, *p* = 0.006) and showed a tendency toward significance when limiting it to the random motion condition (0.344, *p* = 0.058). In the PIVC, prediction accuracy was significant when limiting data to the static condition (0.406, *p* = 0.007) and showed a tendency toward significance when limiting it to the random motion condition (0.328, *p* = 0.083). In V6, pairwise comparisons of prediction accuracy when limiting data to different motion patterns within each ROI revealed significant differences between coherent and random motion patterns (*p* = 0.016) and between coherent motion and static conditions (*p* = 0.0238). Furthermore, PIVC showed a tendency toward significance for the difference in prediction accuracy when limiting data to either coherent motion or static conditions (*p* = 0.063).

### Psychophysical Results

**FIGURE 7:**
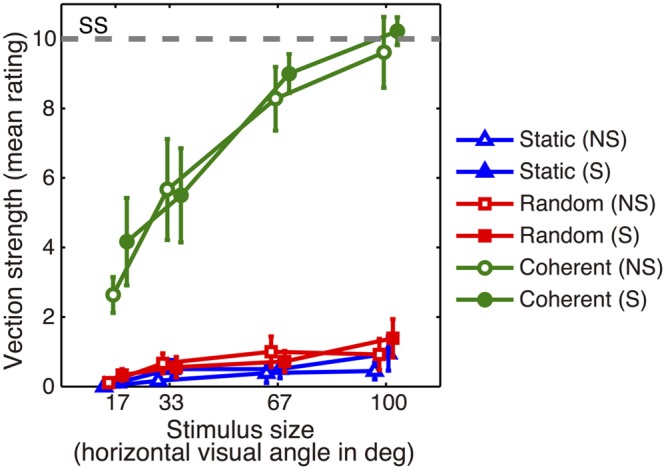
**Results of the psychophysical experiment.** Perceived strength of forward self-motion averaged across nine subjects. Rating scores indicate the strength of subjective self-motion with zero indicating no self-motion and 10 indicating the maximum strength elicited by the standard stimulus (SS, the gray dashed line). Error bars indicate SEMs. S: stereo; NS: non-stereo.

We measured the strength of perceived forward self-motion via magnitude estimation (**Figure 7**), which revealed significant main effects of Motion Pattern (*F*_1.02,8.13_ = 44.2, *p* = 1.15 × 10^-4^), Stimulus Size (*F*_1.55,12.4_ = 38.3, *p* = 1.09 × 10^-5^), and their interaction (*F*_2.20,17.6_ = 26.3, *p* = 3.48 × 10^-6^). Conversely, we found no significant main effect of Stereo Factor or any other interaction. A subsequent *post hoc* analysis revealed the following profiles. For Motion Pattern, the rating score for the coherent motion condition was significantly larger than that of the random motion or static conditions. Additionally, the rating score for the random motion condition was significantly larger than that for the static condition. For Stimulus Size, we found a significantly larger rating score for larger stimuli (except for between the 67° and the 100° conditions) in the coherent motion condition.

### Results of Neuro-perceptual Correlation Analysis

**FIGURE 8:**
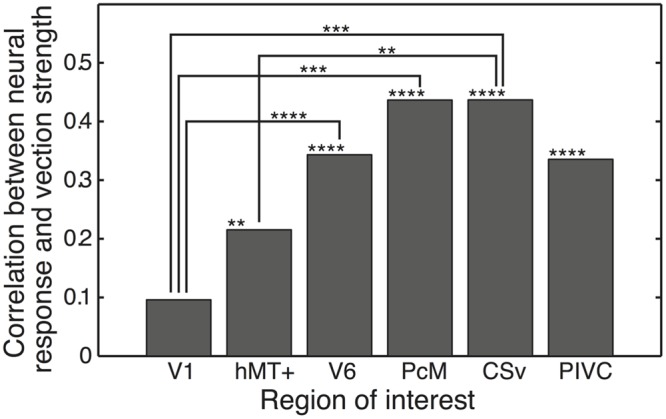
**Results of the correlation analysis.** We computed the neuro-perceptual correlation between neural responses and perceived self-motion strength. Stars and asterisks indicate the statistical significance levels for each ROI and the difference between ROIs. ^∗∗^*p* < 0.01; ^∗∗∗^*p* < 0.001; ^∗∗∗∗^*p* < 0.0001.

We derived the neuro-perceptual correlation between the ROI-averaged activation and the perceived self-motion strength for each ROI (**Figure 8**). We found significant positive correlations in the hMT+ (*r* = 0.215, *p* = 4.39 × 10^-3^), V6 (*r* = 0.343, *p* = 6.84 × 10^-7^), PcM (*r* = 0.437, *p* = 5.52 × 10^-11^), CSv (*r* = 0.437, *p* = 5.29 × 10^-11^), and PIVC (*r* = 0.336, *p* = 1.32 × 10^-6^), but not in V1 (*r* = 0.0957, *p* = 0.479). Subsequent pairwise comparisons of the derived correlation coefficients revealed significant differences between V1 and V6 (*p* = 1.27 × 10^-9^), PcM (*p* = 1.49 × 10^-4^), and CSv (*p* = 6.14 × 10^-4^), and between hMT+ and CSv (*p* = 0.0102). The correlation coefficients for V6, PcM, and CSv were higher than that for V1, and the correlation coefficient for CSv was higher than that for hMT+.

## Discussion

### Differential Response Properties to Visual Self-motion Signals in the Medial Cortical Regions

The results of the conventional SPM (**Table 1**; **Figure 4A**) and univariate ROI analyses (**Table 2**) demonstrated not only a known optic-flow selective response in V6 (Cardin and Smith, 2010; Pitzalis et al., 2010), PcM (Cardin and Smith, 2010), and CSv (Wall and Smith, 2008; Cardin and Smith, 2010), but also a unique inhibitory response to random motion relative to static conditions in the CSv. Furthermore, the multi-voxel analysis revealed that PcM and CSv, but not V6, show a multi-voxel response pattern specific to coherent motion (**Figure 6C**). In contrast, activity in V6 and PcM (partially) successfully classified stimulus size, but that in CSv did not (**Figures 6A,B,D**). In the context of our findings, we discuss the differential involvement of each of the medial optic-flow regions in the processing of visual self-motion signals in the following sections.

#### Visual Self-motion Specific Representation in the PcM and CSv but not V6

The Motion Pattern classification results in the MVPA (**Figures 6B,C**) suggest the presence of visual self-motion specific multi-voxel patterns in the PcM and CSv. In the PcM and CSv, the coherent motion condition was accurately classified, while each of static and random motion conditions was not. Moreover, as the red lines in **Figure 6C** show, direct comparisons between different binary classifications consistently indicated that in the PcM and CSv, but not in any other assessed region, predictions of the differentiation between coherent motion and random motion were significantly more accurate than those between random motion and static conditions as a control. These results suggest that the PcM and CSv not only show activation selective to visual self-motion signals, but that they also exhibit representation specific to such signals. On the contrary, in V6, all three conditions comprising Motion Pattern were classifiable, as was the case for hMT+ and V1. Therefore, although V6 exhibits optic-flow selective activation, this region may not specifically represent visual self-motion signals. Alternatively, this region may have a more general function in processing optic-flow originating from both self- and object-motion. This possibility is further discussed in Section “Visuotopic Representation differs among Medial Optic-Flow Regions.”

#### Inhibitory Effect of Self-motion Incompatible Signal in the CSv

Unlike in other assessed regions, univariate activation in CSv was statistically significantly lower in the random motion condition than in the static condition (**Table 2**), although the difference appears to be small (**Figure 5**). To the best of our knowledge, lower activation in response to random motion stimuli has not previously been reported in any visually responsive cortical areas. This result might reflect an inhibitory effect of a self-motion incompatible signal in CSv. We speculate that visual information incompatible with self-motion (e.g., random dot motion) suppresses activity in the CSv, but that visual stimuli that are self-motion neutral (e.g., static dots) do not. The observed optic-flow selectivity in the CSv for coherent motion (i.e., self-motion compatible stimuli) also supports our interpretation. One might argue that this effect simply arises from the difference in low-level visual features between the stimuli. However, we consider this to be unlikely because the visual features comprising the stimuli were kept identical between the conditions, except for the presence of motion components in the random and coherent motion conditions. Although further clarification is needed, the neural response in CSv likely contains both excitatory and inhibitory components, which are induced by self-motion compatible and incompatible visual inputs, respectively. This combination might serve as a mechanism to enhance the sensitivity of visual self-motion estimation.

Our results regarding the MVPA may also suggest that inhibition by self-motion incompatible signals in the CSv not only affects univariate activation but also multi-voxel response patterns. With respect to Motion Pattern classification in the CSv, we were able to conduct relatively accurate classification for both coherent and random motion, whereas this was the case only for coherent motion in the PcM (**Figure 6B**). This characteristic of the CSv response is consistent with the results of the binary classifications (**Figure 6C**). While the accuracy for coherent vs. random motion tended to be higher than that for coherent motion vs. static stimuli in the CSv, these accuracies were comparable in the PcM. Thus, inhibition via self-motion incompatible signals (e.g., random motion) and activation via self-motion signals (i.e., coherent motion) might induce different multi-voxel patterns in the CSv. However, we cannot deny the possibility that random motion inhibits activation uniformly across most of the voxels comprising the CSv, while at the same time sparsely activating a small number of voxels in this region. Indeed, this could also explain the present univariate and multi-voxel profiles for the CSv. Further investigation is necessary to elucidate the inhibitory effect of self-motion incompatible signals on both univariate and multi-voxel response properties of the CSv.

#### Visuotopic Representation Differs among Medial Optic-flow Regions

Our MVPA results for Stimulus Size classification revealed a significant effect in V6, a partially significant effect in the PcM, and no significant effect in the CSv (**Figure 6D**). Here, we consider the idea that this distinction between V6 and CSv may result from the known retinotopic organization in V6 (Pitzalis et al., 2006) and a possible absence of such retino- or spatio-topic representation in CSv. We hypothesize that if a visual area has clear retinotopic organization, visual stimulation with varying retinal coverage will differentially activate retinotopically corresponding sub-regions within that area, which will eventually result in differential spatial response patterns across stimulus sizes, regardless of any motion pattern. Indeed, not only did the Stimulus Size classification show significantly higher prediction accuracy than chance level in V6 (**Figure 6A**), but also subsequent analysis that performed Stimulus Size classification for each motion pattern showed significant prediction accuracy for each motion pattern (**Figure 6D**). The hMT+, which is also known to have retinotopy (Huk et al., 2002; Amano et al., 2009), had an identical multi-voxel profile to that of V6, despite relatively low discriminability in terms of Stimulus Size (**Figure 6C**). Such high discriminability in V6 might reflect clearer retinotopic organization for the far peripheral visual field in this region compared with that of the hMT+. Previous reports of large representations for visual periphery in V6 (Pitzalis et al., 2006) support this view. In contrast, Stimulus Size was not classifiable by the CSv under any motion pattern, including coherent motion (**Figure 6D**), suggesting that CSv may not have a clear retinotopic representation like that found in V1 and V6. In PcM, Stimulus Size classification was only significant for the coherent motion condition (**Figure 6D**), indicating that PcM partially represents retino- or spatio-topic information, especially when presented in a visual self-motion signal. The direct pairwise comparisons among the medial regions for Stimulus Size classification consistently showed that prediction accuracy for V6 was significantly larger than that for PcM or CSv, and that accuracy for PcM was significantly larger than that for CSv (**Figure 6A**).

Here, one might argue that inability of CSv to accurately classify Stimulus Size is simply because the univariate response amplitude is low, which may bias the resulting SVM prediction accuracy (Smith et al., 2011). To assess this possibility, we computed the average univariate response amplitude across all experimental conditions for each ROI (Supplementary Figure S3). We found that PIVC, which exhibited a significant Stimulus Size classification performance for the static condition, showed an averaged univariate response amplitude that did not significantly differ from that of the CSv. Thus, we consider it unlikely this characteristic of the CSv can be totally explained by a low response amplitude *per se*.

#### Differential Roles for Medial Optic-flow Regions in Self-motion Processing

The differential response properties among medial optic-flow regions discussed in the above section, together with previous studies, may further suggest a distinct role for each of these regions in the processing of optic-flow. The retinotopically organized visual response observed in V6, but not as clearly in PcM or CSv, may suggest that V6 represents not only self-motion but also object-motion in the sense that the retinal position of the stimulation informs the spatial location of its source, i.e., a moving object. This conforms to studies that have proposed that V6 may play a pivotal role in parsing complex retinal motion into self- and object- motion components (Arnoldussen et al., 2013) or in providing information about objects’ locations in space during self-motion (Pitzalis et al., 2013). In contrast, the presence of multi-voxel pattern specific to visual self-motion and the less clear retinotopically organized response in CSv may suggest its involvement in the processing of self-motion rather than object-motion. CSv has been reported to show its ability to integrate eye movements with retinal motion (Fischer et al., 2012) and strong vestibular response (Smith et al., 2012). Along with this evidence, our results showing the unique inhibitory effect of self-motion incompatible stimuli and an optic-flow specific multi-voxel response pattern support the view that CSv is significantly involved in encoding self-motion (Wall and Smith, 2008; Cardin and Smith, 2010), or more specifically, in integrating different sources of information into a multimodal representation of self-motion (Arnoldussen et al., 2013). Furthermore, CSv might be coding different types of self-motion signals, including forward self-motion. Indeed, the CSv has been reported to exhibit multi-voxel response patterns with which leftward and rightward heading direction changes were successfully decoded (Furlan et al., 2013). This idea is supported by previous investigations that reported CSv exhibits sensitivity to different types of optic-flow patterns (expansion: Wall and Smith, 2008; Cardin and Smith, 2010; Fischer et al., 2012; Arnoldussen et al., 2013; rotation: Pitzalis et al., 2013; translation: Fischer et al., 2012; Pitzalis et al., 2013), although a direct investigation is needed. In contrast, the involvement of PcM in self-motion processing might rely mostly on visual input. Studies have conjectured a variety of functional roles for the precuneus cortex including visuo-spatial imagery, episodic memory retrieval, self-processing, and consciousness (for a review, see Cavanna and Trimble, 2006). The response properties of PcM identified by our study (e.g., optic-flow selectivity with partial retino- or spatio-topic representation) could theoretically reflect modulated processing related to internally generated visuo-spatial imagery by externally initiated visual self-motion processing (e.g., updates of surrounding environmental imagery facilitated by self-motion estimated from externally generated sensory inputs). These assessments require further investigation.

### Perception of Forward Self-motion under Wide-view Stereoscopic Stimulation

Our psychophysical experiment confirmed that expanding optic-flow can induce subjective ratings of perceived forward self-motion strength that are significantly higher for coherent motion than for random motion or static conditions (**Figure 7**). We also observed an effect of stimulus size on this rating, which systematically increased as a function of stimulus size under the coherent motion condition (**Figure 7**). The effect of stimulus size has been well-documented for rotational and translational optic-flow stimuli (e.g., Nakamura and Shimojo, 1998; Tarita-Nistor et al., 2006), but has not, to the best of our knowledge, been systematically examined for expanding optic-flow. Thus, our psychophysical results may represent new evidence demonstrating the effect of stimulus size on visually induced forward self-motion perception.

In contrast, the previously reported effect of stereoscopic vs. non-stereoscopic stimulation on perceived self-motion strength (Palmisano, 1996, 2002) was not significant in our experiment (**Figure 7**). We speculate that this may be related to the stimulus duration, which was 1 min or longer in previous studies (Palmisano, 1996: 3 min or 90 s; Palmisano, 2002: 60 s) and considerably shorter (3 s) in ours. This short duration was the result of fMRI design constraints (a large number of experimental conditions) and a limited scanning-run duration. Palmisano (1996) suggested that stereoscopic cues may directly facilitate forward vection by providing motion-in-depth information, and indirectly facilitate forward vection by disambiguating the depth order of objects in the optic-flow. This view might explain the absence of the stereo effect in our study, in which the depth order of the random dots was unambiguously provided by the binocular disparity gradient. Thus, the direct effect of stereoscopic cues may require longer stimulus durations than those used in our study, explaining why we did not observe a distinction between stereo and non-stereo conditions. The psychophysical results in Arnoldussen et al. (2013) also support this idea. While they found no significant stereo effect on vection strength when the duration of optic-flow presentation was short (2 s), they did observe an effect when the depth order of the random dots was made ambiguous by introducing noise components. Therefore, we believe that the absence of the stereo effect in our study might be due to the short stimulus duration, although further direct investigation is required for clarification.

When following the conventions of psychophysical measurements of vection, one might argue that the subjective self-motion rating in our study does not purely reflect vection ‘strength,’ which is typically measured during the presence of vection that arises after a ‘latency’ period. Because of the short stimulus duration and the instructions given in our study, subjects rated the strength of self-motion for the entire duration of each stimulus presentation (3 s), and did not discriminate between vection and non-vection periods. Thus, the measured rating might include the effect of both vection strength and vection latency. We needed this measure to directly compare the subjective self-motion strength and the corresponding neural activation, both of which were elicited during identical stimulus presentation periods. As latency and strength are known to have a consistent relationship (i.e., latency is typically short when strength is high), we consider the measure used in our study to be acceptable as a subjective measure of self-motion strength that represents a mixture of conventional vection latency and strength measures.

### Neuro-perceptual Correlation of Subjective Self-motion Strength

Our neuro-perceptual correlation analysis identified a significant positive correlation between subjective self-motion strength and univariate activation in the hMT+, V6, PcM, CSv, and PIVC in a naturalistic viewing condition (i.e., wide-view stereoscopic stimulation). Subsequent pairwise comparisons revealed that the neuro-perceptual correlation was significantly higher for V6, PcM, and CSv than for V1, and for CSv compared with hMT+ (**Figure 8**). As V1 did not show optic-flow selectivity in its activation or spatial response pattern (**Table 2** and **Figure 6**), the higher neuro-perceptual correlation observed in the V6, PcM, and CSv indicates that these areas are more likely than the hMT+ or PIVC (which did not differ from V1) to be implicated in neural circuits that give rise to the perception of self-motion. More specifically, among the medial optic-flow regions (i.e., V6, PcM, and CSv), CSv is most likely to represent perceived self-motion strength compared with the other assessed regions because it was the only region that exhibited higher neuro-perceptual correlation compared with hMT+.

It can be argued that the neuro-perceptual correlation in the CSv is merely the result of an artifact of stimulus size potentially arising in a region with a visual spatial representation (e.g., retinotopy). However, as discussed above, CSv exhibited a correlation coefficient significantly higher than that of V1, a visual area well-known to have clear retinotopic organization. Furthermore, our MVPA results indicated that the CSv may not have a distinct retino- or spatio-topic representation, such as that observed in V1 (see Differential Response Properties to Visual Self-Motion Signals in the Medial Cortical Regions). Therefore, we consider it unlikely that the neuro-perceptual correlation in the CSv is derived solely by an artifact of stimulus size. We instead suggest that the activation intensity itself correlates with the percept.

Here, based on the findings of our study, we try to interpret the inconsistency found in previous studies regarding the neuro-perceptual correlation of self-motion. Brandt et al. (1998) and Becker-Bense et al. (2012) used an experimental paradigm that was similar to that of the present study to quantitatively assess the neuro-perceptual correlation of self-motion. However, their results critically differed from those of our study. While significant positive neuro-perceptual correlation was identified in the three medial optic-flow regions (i.e., V6, CSv, and PcM) in our study, only the parieto-occipital region nearby V6 was identified in Brandt et al. (1998), and no medial cortical region in Becker-Bense et al. (2012). We speculate that this difference might be caused by the limiting coarse spatial resolution of Positron emission topography (PET) used by Brandt et al. (1998) and Becker-Bense et al. (2012). The use of relatively high spatial resolution of fMRI combined with wide-view visual stimulation in our study may have enabled us to identify positive neuro-perceptual correlation in the medial optic-flow regions. This is supported by an analysis presented in Supplementary Table S1 and Figure S1. We computed a parametric map of neuro-perceptual correlation of subjective self-motion strength for each stimulus size condition. Consequently, we identified the positive correlation in medial optic-flow regions V6, PcM, and CSv only in the 100° condition but not in the other smaller stimulus size conditions (Supplementary Table S1 and Figure S1). This result suggests that the combination of wide-view visual stimulation and high spatial resolution of fMRI might be necessary to robustly identify neuro-perceptual correlation of self-motion perception in PcM and CSv. In particular, CSv was conjointly identified as a region to have representation specific to optic-flow in our MVPA results.

In contrast to the studies discussed above, Kleinschmidt et al. (2002) and Kovács et al. (2008) adopted a different experimental paradigm, in which they compared activation during vection with that during non-vection periods under continuous optic-flow stimulation. Their vection vs. non-vection contrast has identified multiple cortical regions that overlap but are wider than optic-flow sensitive areas, including the early visual cortex and the cerebellum. However, the only region commonly identified was the hMT+, which showed activation in Kovács et al. (2008) but inhibition in Kleinschmidt et al. (2002). Conversely, both Kovács et al. (2008) and our analyses commonly identified a positive effect in the hMT+ and precuneus, although the two studies differed in terms of experimental paradigm and the stimulus size used (Kovács et al., 2008: 30° in diameter; our study: 100° × 67.7°). Both Kovács et al. (2008) and our study used an expansion pattern, while Kleinschmidt et al. (2002) used a rotation pattern. Thus, the stated inconsistency might be because of the difference in the type of optic-flow used. Here, by extending the concept of reciprocal inhibitory visuo-vestibular interaction originally developed by Brandt et al. (1998), we speculate that in Kleinschmidt et al. (2002), the absence of vestibular information, which indicated no self-rotation, had an inhibitory effect on the hMT+ response to visual rotational pattern, which conversely indicated a continuous angular acceleration. In Kovács et al. (2008) and in our study, the expansion patterns were consistent with the vestibular information because both indicated zero acceleration, thus avoiding such a visuo-vestibular conflict. Although the previously reported evidence regarding vestibular modulation on hMST (Smith et al., 2012) supports this notion, further investigation is needed to clarify under what conditions the reciprocal inhibitory visuo-vestibular interaction occurs, including assessments using different types of optic-flow under different experimental paradigms.

## Conclusion

The present fMRI study revealed differential univariate and multi-voxel response properties to visual self-motion signals among the following medial optic-flow regions: V6, PcM, and CSv. Along with optic-flow selective univariate activation in these medial regions under wide-view stereoscopic stimulation, we found a unique inhibitory effect of self-motion incompatible signals in the CSv. Furthermore, our MVPA results demonstrated a multi-voxel response pattern specific to visual self-motion signals in the PcM and CSv, but not in V6. Conversely, stimulus size had a strong, partial, and absent effect on the multi-voxel pattern in V6, the PcM, and the CSv, respectively, which may reflect the known retinotopic representation in V6 and the absence of clear visuospatial representation in the CSv. Besides, the neuro-perceptual correlation for self-motion was significantly higher for V6, the PcM, and the CSv when compared with V1, and higher for the CSv when compared with the visual motion area hMT+. These results convergently suggest the important involvement of the medial optic-flow regions, especially the CSv, in visual self-motion processing that may give rise to its percept.

## Author Contributions

All authors listed, have made substantial, direct and intellectual contribution to the work, and approved it for publication.

## Conflict of Interest Statement

The authors declare that the research was conducted in the absence of any commercial or financial relationships that could be construed as a potential conflict of interest.

## Footnotes

1 http://freesurfer.net

2 http://marsbar.sourceforge.net
