# Supplementary material for: Differential Responses to a Visual Self-Motion Signal in Human Medial Cortical Regions Revealed by Wide-View Stimulation
Source: Front Psychol. 2016 Mar 4;7:309. doi: 10.3389/fpsyg.2016.00309 (PMC4777731; doi:10.3389/fpsyg.2016.00309)
Supplement: Supplementary file 1 [file Table_1.DOCX]

**Table S1. Significant clusters for the positive parametric effect of subjective self-motion strength (eight subjects).** For each stimulus size condition, we searched for cortical regions that showed statistically significant parametric effect of subjective self-motion strength. A parametric modulation analysis was applied to the main experimental data for the eight subjects that underwent the psychophysics experiment. We used a preprocessing procedure identical to that of the SPM analysis (see Section 2.5.1 in the main text). We created a parametric predictor of subjective self-motion strength for each stimulus size condition by assigning the corresponding psychophysical rating of self-motion strength to each stimulus presentation period. We identified clusters of statistically significant contiguous parametric effect (height thresholds: uncorrected p<0.001 at peak level, p<0.05 FDR-corrected at cluster level) in 17° and 100° but none in 33° or 67° conditions. The coordinate of the peak location of each cluster is provided in Montreal Neurological Institute stereotaxic space.

| Region name | | Laterality | Peak location | | |  | Peak val. | Size |
| --- | --- | --- | --- | --- | --- | --- | --- | --- |
|  |  |  | x | y | z |  | (Z-score) | (mm^3^) |
| **(1) Parametric modulation of vection strength (100°)** | | | | | | | | |
|  | Cingulate sulcus (CSv) | L | -14 | -20 | 42 |  | 5.35 | 648 |
|  |  | R | 16 | -26 | 44 |  | 4.48 | 376 |
|  | Precuneus (PcM) | L | -18 | -40 | 46 |  | 4.76 | 608 |
|  |  | R | 8 | -46 | 54 |  | 4.30 | 248 |
|  | Parieto-occipital sulcus (V6) | R | 16 | -78 | 40 |  | 4.35 | 288 |
| **(2) Parametric modulation of vection strength (67°)** | | | | | | | | |
|  |  | No significant cluster | | | | | | |
| **(3) Parametric modulation of vection strength (33°)** | | | | | | | | |
|  |  | No significant cluster | | | | | | |
| **(4) Parametric modulation of vection strength (17°)** | | | | | | | | |
|  | Inferior parietal lobule | R | 40 | -42 | 56 |  | 3.95 | 432 |
