# Supplementary figures and images for: Differential Responses to a Visual Self-Motion Signal in Human Medial Cortical Regions Revealed by Wide-View Stimulation
Source: Front Psychol. 2016 Mar 4;7:309. doi: 10.3389/fpsyg.2016.00309 (PMC4777731; doi:10.3389/fpsyg.2016.00309)

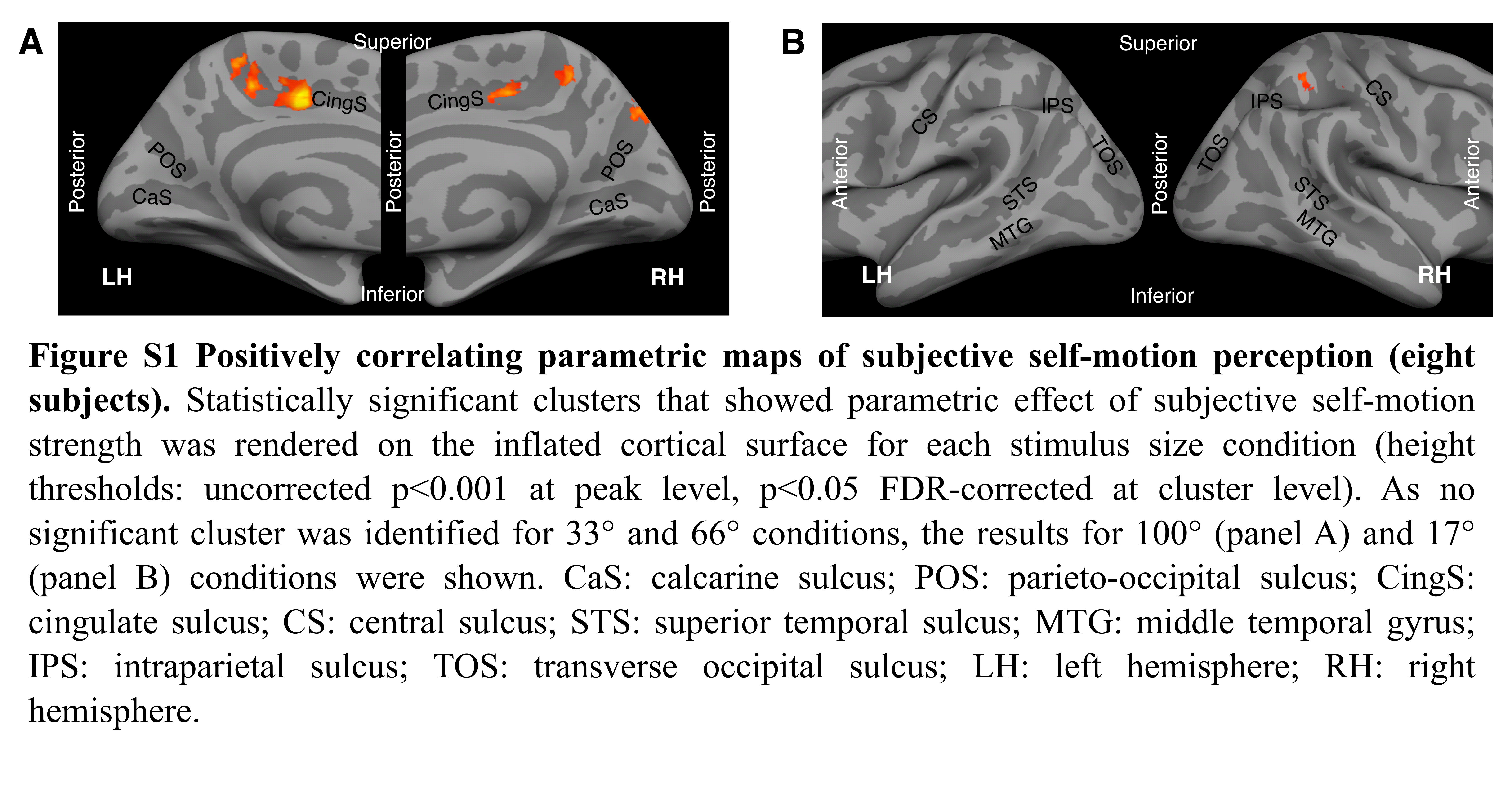

Supplement: Supplementary file 2 [file Image_1.TIFF]

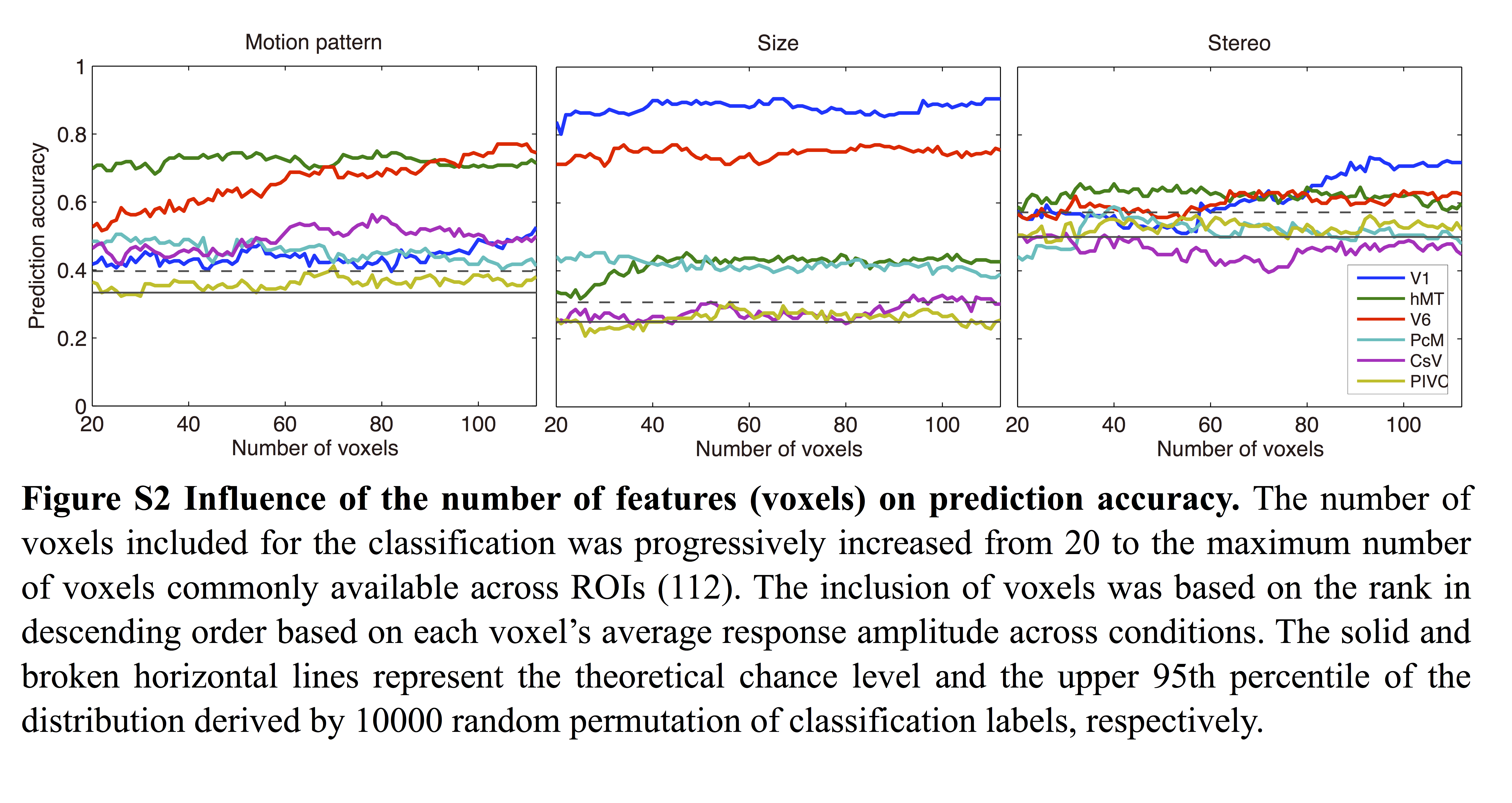

Supplement: Supplementary file 3 [file Image_2.TIFF]

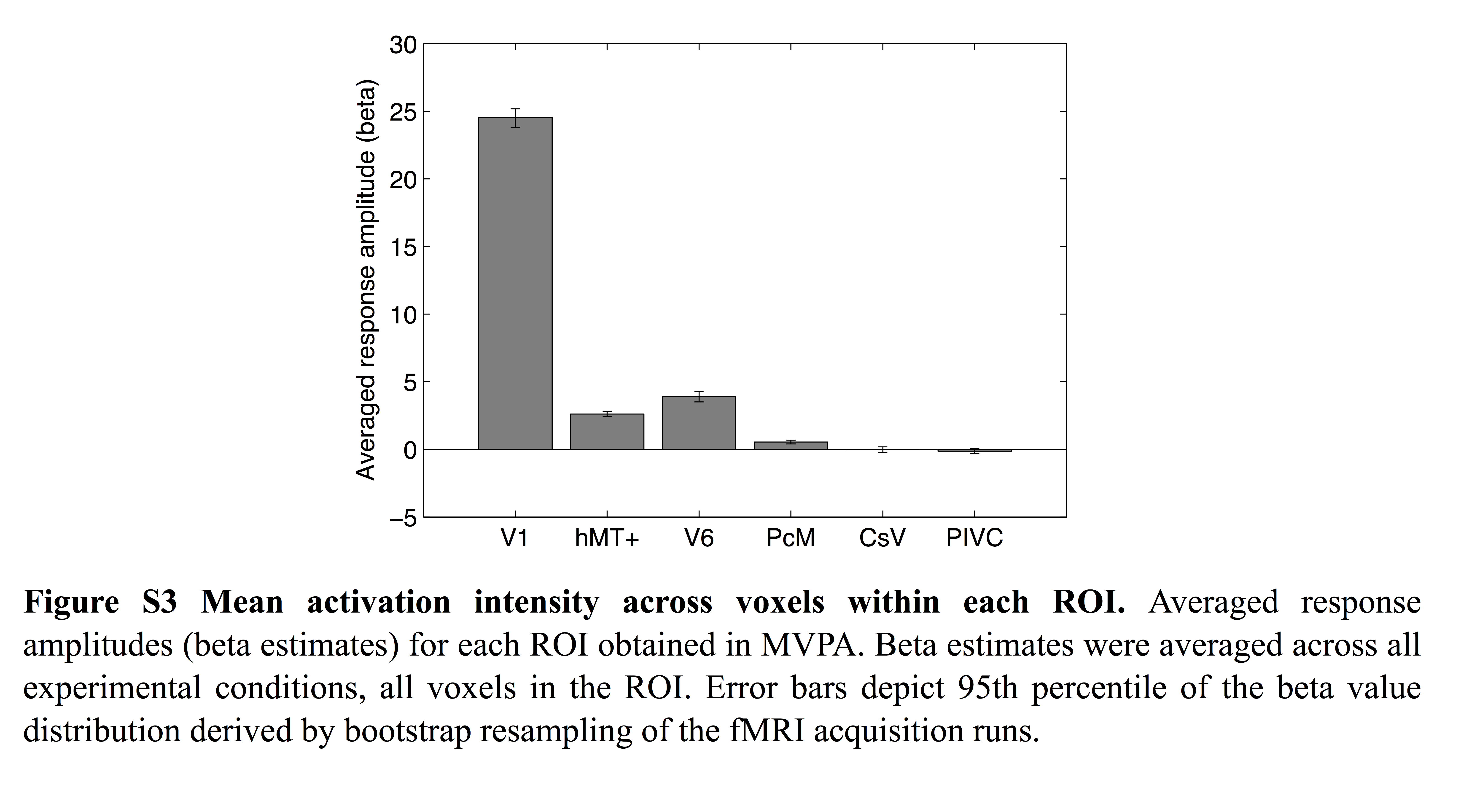

Supplement: Supplementary file 4 [file Image_3.TIFF]
